# Dermal Concentration Versus Systemic Bioavailability of Topical Lidocaine and Tetracaine: An Exploratory Pharmacokinetic Pilot Study in Göttingen Minipigs

**DOI:** 10.3390/pharmaceutics18010040

**Published:** 2025-12-28

**Authors:** Paweł Biernat, Dawid Bursy, Dominik Marciniak, Konrad Krajewski, Jan Meler, Radosław Balwierz

**Affiliations:** 1 Department of Drug Forms Technology, Faculty of Pharmacy, Wrocław Medical University, 211 Borowska St., 50-556 Wroclaw, Poland; pawel.biernat@umw.edu.pl (P.B.);; 2 Biotts SA, Wroclawska 44c St., Bielany Wroclawskie, 55-040 Wroclaw, Poland; 3 Faculty of Computer Science and Management, Wroclaw University of Science and Technology, 50-370 Wroclaw, Poland; 4 Institute of Chemistry, University of Opole, Oleska 48 St., 45-052 Opole, Poland

**Keywords:** lidocaine, tetracaine, bioavailability, pharmacokinetics, Göttingen minipig, LC–MS/MS, formulation effects

## Abstract

**Background:** Lidocaine, classified as an amide-type agent, and tetracaine, designated as an ester-type agent, are frequently co-formulated for dermatologic procedures. Despite the extensive literature on the pharmacokinetics (PK) of these substances, there is a paucity of head-to-head comparisons of intravenous (IV) and topical administration in the same preclinical model. Absolute bioavailability (F%) is imperative for optimizing formulation design and safety. **Methods:** A single-dose, single-sequence, three-period pilot study was performed in male Göttingen mini-pigs. The first period of the study involved the intravenous bolus administration of lidocaine HCl and tetracaine HCl, with a dosage of 1 mg/kg for each agent. In Period 2, the topical application of Pliaglis (a combination of 7% lidocaine and 7% tetracaine, with a concentration of 10 g/100 cm^2^ and a duration of 60 min) was utilized. In Period 3, the pharmacokinetic profile of Z4T4L4 (a formulation comprising 4% lidocaine HCl and 4% tetracaine HCl) was assessed under the same experimental conditions. Blood samples were collected up to 24 h after the administration of the drug; skin biopsies were obtained 90 min after the application of the test substance. Plasma and skin concentrations were measured by means of validated liquid chromatography–tandem mass spectrometry (LC–MS/MS). PK parameters were derived using a noncompartmental analysis approach, while F% was calculated through AUC comparison with IV dosing. **Results:** Subsequent to intravenous administration, the mean elimination half-lives of lidocaine and tetracaine were determined to be 1.62 h and 1.85 h, respectively. Pliaglis demonstrated higher skin concentrations of lidocaine (358 μg/g) and tetracaine (465 μg/g) compared to Z4T4L4 (33.6 μg/g and 46.1 μg/g, respectively). Despite lower skin levels, Z4T4L4 produced higher F% (lidocaine: 1.98% vs. 1.41%; tetracaine: 3.34% vs. 1.26%). The time to maximum plasma concentration (T_max_) for lidocaine was found to be 2–4 h (Pliaglis) and 2–8 h (Z4T4L4), while for tetracaine, it was 1–8 h (Pliaglis) and 2–8 h (Z4T4L4). **Conclusions:** In this preliminary study, which included three subjects, Z4T4L4 exhibited a numerical tendency towards increased systemic bioavailability in comparison with Pliaglis. This observation was noted despite the fact that Z4T4L4 resulted in markedly lower skin concentrations. Due to the exploratory nature of the pilot study (*n* = 3), observed differences are reported as numerical trends. The data suggest that Z4T4L4 may enhance systemic absorption while reducing skin retention, highlighting a potential formulation-dependent dissociation between local concentration and systemic bioavailability. These preliminary findings provide in vivo evidence of a divergence between eutectic-based tissue retention and enhancer-driven systemic flux. This highlights that formulation design fundamentally dictates the safety profile of local anesthetics, necessitating a balance between local efficacy and systemic safety.

## 1. Introduction

Lidocaine and tetracaine are local anesthetics that are classified as amide- and ester-type compounds, respectively. These agents function by obstructing voltage-gated sodium channels, thereby impeding nerve conduction and inducing analgesia [1,2,3]. A comparison of tetracaine and lidocaine reveals distinguishing characteristics in their binding properties and kinetic interactions with sodium channels. The predominant ionized form of tetracaine, through the hydrophilic pathway, binds within the channel pore, thereby stabilizing the inactivated state and inducing a more potent and prolonged conduction block [4,5,6,7]. Clinically, lidocaine is widely used in dermatological procedures, laser treatments, and minor surgery, as well as in the topical management of chronic pain, diabetic neuropathy, carpal tunnel syndrome, and lower back pain. The primary field of application for tetracaine is dermatology, particularly in combination with lidocaine in topical anesthetic formulations [8,9]. In 2006, the FDA approved a fixed 7%/7% lidocaine–tetracaine cream (Pliaglis) for superficial dermatological procedures. A meta-analysis of randomized controlled trials confirmed that the application of patches or creams containing this combination results in significantly greater analgesia than placebo after 30–60 min of application [10]. The two agents differ substantially in their pharmacokinetic behavior. Lidocaine has a half-life of 1.5–2.0 h following intravenous administration in healthy adults [11,12]. Its apparent clearance has been shown to average 48 L/h when applying two or fewer patches, increasing to 67 L/h with three patches [11,12]. However, oral bioavailability is constrained by substantial first-pass metabolism, while absorption after vaginal administration is dose-proportional with no systemic accumulation [13]. Conversely, tetracaine is subject to rapid hydrolysis by plasma esterases, resulting in a significantly diminished half-life [14,15,16,17,18]. Its metabolic processes are predominantly facilitated by ester hydrolysis, a process that is catalyzed by pseudocholinesterase, resulting in a notably elevated clearance rate. The systemic bioavailability of tetracaine following topical administration is widely regarded as negligible, thereby supporting its favorable safety profile [7,14] provided that local concentrations reach therapeutic thresholds within the dermis. Furthermore, lipophilicity exerts a significant influence on their disposition, with lidocaine exhibiting moderate lipophilicity, thereby contributing to its penetration and duration of action [2]. In contrast, tetracaine demonstrates a greater degree of lipophilicity, which favors membrane permeation and prolonged effect [19]. The topical route offers both agents the advantage of local delivery with minimal systemic exposure [14,20].

For topical anesthetics, plasma concentrations alone do not fully reflect efficacy, since the therapeutic target lies within the cutaneous layers [21]. Cutaneous pharmacokinetic studies have demonstrated that maximum concentrations (C_max_) in skin compartments may differ substantially from plasma levels [22]. Recent advances have focused on enhancing the dermal absorption of local anesthetics through novel carriers [18,23,24]. In this context, a pharmaceutical preparation was developed containing 4% lidocaine hydrochloride and 4% tetracaine hydrochloride (patents No. PL245545B1 [25] and US12201621B2 [26]).

The selection of an appropriate preclinical model is of critical importance for the acquisition of pharmacokinetic data that is relevant to clinical applications. The Göttingen minipig is a widely utilized model due to its anatomical and structural similarities to human skin, as evidenced by studies [27,28,29]. The drug’s metabolic profile in vivo exhibits significant similarity to that of humans, thereby supporting its utilization in dermal absorption and cutaneous pharmacokinetic investigations. A substantial body of research has previously validated the minipig as a suitable model for dermatological formulations and wound-healing research. Histological analyses have confirmed the reproducibility of regenerative processes [30].

Despite extensive clinical use of lidocaine and tetracaine, key gaps remain in their comparative pharmacokinetic characterization. The majority of published studies have examined single compounds or a single administration route, thereby preventing head-to-head evaluation of pharmacokinetics across routes [31,32,33]. The presence of methodological variability, the utilization of relatively small study populations, and the employment of disparate analytical approaches serve to further restrict the comparability of the studies. The paucity of data regarding the absolute bioavailability of tetracaine is primarily attributable to its plasma instability and the attendant analytical challenges [31,34]. Furthermore, since the approval of Pliaglis, there have been few head-to-head comparisons in minipigs with IV versus topical within-subject designs [8,35].

To address these gaps, the present study evaluated the single-dose pharmacokinetics of lidocaine hydrochloride and tetracaine hydrochloride following intravenous and topical administration in male Göttingen minipigs. The present study investigated a novel 4%/4% formulation in conjunction with Pliaglis. The objective of this study is threefold: firstly, to establish intravenous reference parameters for absolute bioavailability calculations; secondly, to quantify the bioavailability of both agents after topical dosing as a surrogate marker for transdermal flux efficiency [20,22]; and thirdly, to compare the dermal delivery mechanisms (e.g., depot formation vs. systemic clearance) of the two formulations through plasma pharmacokinetics and skin tissue concentrations. The objective of these findings is twofold: first, to inform the optimization of lidocaine/tetracaine product dosages; and second, to enhance the comprehension of pharmacokinetic disparities between amide- and ester-type anesthetics across various administration pathways.

## 2. Materials and Methods

### 2.1. Animals

Male Göttingen minipigs were procured from Ellegaard Göttingen Minipigs (Sorø Landevej 302, DK-4261 Dalmose, Denmark). At the initiation of the study, the animals were approximately eight months of age, with body weights ranging from 16 to 20 kg. The target range for dosing was established at 18–22 kg, corresponding to an age range of 8–10 months. The Göttingen minipig was selected as the experimental model due to the close histological and functional resemblance of its skin to human skin, as previously described by Biernat [36] and Todo [37]. Three animals (*n* = 3) were enrolled in the study. This number was determined in compliance with the 3Rs principles (Reduction, Refinement, Replacement) to minimize animal use while maintaining scientific validity. A fixed-sequence, within-subject, three-period design was implemented, thereby enabling each animal to serve as its own control and thereby reducing inter-individual variability.

The in-life phase and bioanalytical analysis were conducted at an independent, GLP-compliant accredited facility, ensuring data integrity and impartial execution. The protocol was reviewed and approved on 19 November 2019, by the Animal Welfare Body under project license AVD2360020172866, which was issued by the Central Authority for Scientific Procedures on Animals (CCD). This approval was granted in accordance with the Dutch Animal Experiments Act (December 2014). The study was reported in accordance with the ARRIVE guidelines.

The animals were not subjected to necropsy following the completion of the study, which would have allowed for their potential inclusion in future research. The subjects were identified through the use of ear tags, and cages were marked with color-coded identification cards. The animals were sourced from the facility’s breeding colony and had been acclimated to the toxicology housing conditions prior to enrolment.

The housing was composed of anodized aluminum or stainless-steel cages with sawdust bedding. The environmental parameters were meticulously maintained at temperatures ranging from 18 to 24 °C (averaging 21 °C), relative humidity levels ranging from 30 to 70% (averaging 49–53%), a 12 h light/dark cycle with nighttime illumination, and a minimum of 10 air changes per hour. Animals were provided with a commercial SDS diet for minipigs twice daily, and tap water was supplied ad libitum via an automated watering system. Throughout the duration of the study, veterinary care was provided consistently.

### 2.2. Study Design

The objective of the present study was to compare the bioavailability and pharmacokinetic profiles of lidocaine and tetracaine following topical application of the reference product (Pliaglis cream) and a novel formulation (Z4T4L4). A single-dose, single-sequence, three-period longitudinal design was employed in one group of three male Göttingen minipigs (*n* = 3). The experimental phase was conducted between 21 November 2019, and 20 March 2020, immediately following the ethical approval granted on 19 November 2019. The experimental subjects were administered the three treatments in question, with washout intervals between each period of administration to avoid carryover effects.

The first period (intravenous) is characterized by the following: Lidocaine hydrochloride and tetracaine hydrochloride were administered as a slow bolus via the ear vein at a dosage of 1 mg/kg each over a period of approximately 1 min. Correction factors of 1.23 for lidocaine and 1.14 for tetracaine were applied to account for the hydrochloride salt mass. The solutions were prepared at a concentration of 2 mg per mL (mg/mL) and administered at a dose of 0.5 mL/kg.

The second period of treatment consisted of the application of topical Pliaglis. A dose of 10 g/100 cm^2^ of Pliaglis (7% lidocaine, 7% tetracaine) was applied to a shaved area on the right flank for a duration of 60 min.

The third period of the study involved the topical administration of the investigational formulation (Z4T4L4). A dose of 10 g/100 cm^2^ of Z4T4L4 (4% lidocaine hydrochloride, 4% tetracaine hydrochloride) was applied to the left flank under the same regimen. Following a 60 min period, the residual cream was removed, and the animals were fitted with protective collars to prevent interference with the application sites until the final blood collection.

The washout periods were one week between Periods 1 and 2, and two weeks between Periods 2 and 3. This design enabled each animal to serve as its own control, thereby reducing inter-individual variability. The clinical observations encompassed mortality, general condition, neurological signs (lethargy, tremors, ataxia), and local skin reactions (erythema, crusting, edema).

A summary of the dosing regimen is presented in Table 1.

### 2.3. Drug Formulations and Doses

Three formulations were evaluated in this study: intravenous solutions of lidocaine hydrochloride and tetracaine hydrochloride, the reference topical cream Pliaglis, and the test formulation Z4T4L4.

Intravenous solutions (Period 1): Lidocaine hydrochloride (batch no. 170947, Fagron, Kraków, Poland) and tetracaine hydrochloride (batch no. 180187, Fagron, Poland) were dissolved in saline (Eurovet Animal Health, Bladel, The Netherlands). Solutions were freshly prepared on the day of dosing (*w*/*v*), homogenized to achieve uniformity, and stored in septum-sealed containers until administration. Each compound was administered at a dose of 1 mg/kg as a slow bolus into the ear vein over a period of approximately 1 min.

In Period 2, the reference product Pliaglis cream (7% lidocaine, 7% tetracaine; batch no. 8469002, Egis Pharmaceuticals, Budapest, Hungary) was applied topically. In Period 3, the test product Z4T4L4 cream (4% lidocaine hydrochloride, 4% tetracaine hydrochloride; batch no. 0011022019, Biotts SA, Wroclaw, Poland; patent No. PL245545B1 [25] and US12201621B2 [26]) was used. The investigational product, Z4T4L4, is a topical hydro-alcoholic formulation containing Lidocaine HCl (4% *w*/*w*) and Tetracaine HCl (4% *w*/*w*). It comprises a synergistic mixture of volatile alcohols (e.g., ethanol), diols (e.g., propylene glycol), and fatty acid esters acting as sorption promoters. This specific composition is designed to reversibly disrupt the stratum corneum lipid organization and increase the thermodynamic activity of the active ingredients upon application, preventing crystallization on the skin surface. These components are designed to temporarily alter the lipid composition of the stratum corneum, thereby enhancing the permeation of the active principles. Both topical formulations were applied at a dose of 10 g/100 cm^2^ (corresponding to 100 mg formulation/cm^2^) to the dorsal region. Hair clipping was performed 48 h prior to dosing to allow skin recovery from potential micro-abrasions, with a final trim performed immediately before dosing if necessary. The skin was cleaned with lukewarm tap water and a neutral liquid soap; no organic solvents or disinfectants (e.g., alcohol) were used during formulation application and removal to prevent skin delipidization or API recrystallization artifacts. Formulations were applied using a disposable plastic spatula and spread in a uniform layer by gentle inunction. After the 60 min exposure period, the residual formulation was removed using the same washing procedure (lukewarm water and neutral soap) and gently dried with paper towels. The amount of residual formulation removed was not quantified in this pilot study.

### 2.4. Sample Collection

The sample collection included both blood and skin biopsies. Blood samples (2.0 mL) were obtained from the jugular vein using K_2_EDTA as an anticoagulant. Immediately following collection, samples were stored on ice and subjected to centrifugation within 2 h at approximately 2000× *g* for 10 min at a temperature of 4–8 °C. Plasma was separated, transferred into two uniquely labeled polypropylene tubes (designated as Aliquots A and B), and frozen on dry ice or stored at −75 °C. Due to the instability of tetracaine in minipig plasma, resulting in a 35.5% decrease in concentration within 1.5 h at room temperature, it was necessary to acidify the plasma with 1% formic acid prior to processing. The plasma (396 μL) was treated with 4 μL of 100% formic acid, yielding a final concentration of 1% *v*/*v*.

The blood sampling schedule is outlined in Table 2. For each animal, 2 mL of sample was obtained at each designated time point.

Skin biopsies were obtained in Periods 2 and 3 from the treated flank 90 min after the initiation of application. The surgical procedure was performed under general anesthesia. Subsequent to the induction process (which occurred approximately one hour after the commencement of the procedure), the surgical site was meticulously cleansed and disinfected using Hibiscrub and alcohol, rinsed with sterile water, and dried thoroughly. This antisepsis step (Hibiscrub and alcohol) was performed only immediately prior to biopsy, after complete removal of the formulation and rinsing; therefore, it was not part of the formulation removal procedure and was applied identically across periods. Biopsies measuring 5 mm in diameter were meticulously excised using instruments manufactured by Servoprax GmbH (Wesel, Germany). Samples were immediately snap-frozen in liquid nitrogen and stored at ≤−75 °C until analysis.

### 2.5. Bioanalytical Methods

The quantification of lidocaine hydrochloride and tetracaine hydrochloride in plasma and skin samples was performed using liquid chromatography coupled with tandem mass spectrometry (LC–MS/MS).

The instability of tetracaine in plasma from minipigs was previously documented, exhibiting a 35.5% reduction within 1.5 h when stored on ice (35.5% decrease within 1.5 h on ice). To mitigate the effects of tetracaine, the plasma underwent acidification with 1% formic acid before processing (4 μL of 100% formic acid per 396 μL plasma; final concentration: 1% *v*/*v*). Acidified samples underwent protein precipitation with acetonitrile, followed by centrifugation. The resulting mixture was then diluted with an appropriate solvent and subjected to analysis. Values below the lower limit of quantification (LLOQ; 0.5 ng/mL for lidocaine, 0.1 ng/mL for tetracaine) were reported as “below LLOQ” (BLQ) and set to zero for summaries and plots; sub-LLOQ values were not graphed.

Skin biopsies were stabilized by immediate snap-freezing in liquid nitrogen and storage at ≤–75 °C. Prior to analysis, samples were homogenized in extraction solvent (12 μL/mg tissue) using a Precellys Evolution bead homogenizer (Bertin Instruments, Montigny-le-Bretonneux, France). Then, the samples were centrifuged, and the supernatant was subjected to LC–MS/MS. The lowest quantifiable amount (LLOQ) of both compounds in skin tissue was determined to be 3.25 μg/g.

The analyses were performed on an API 5000 MS/MS system (Sciex, Framingham, MA, USA) that featured a Turbo Ion Spray interface operating in positive ionization mode and multiple reaction monitoring (MRM) capabilities. The monitored ion transitions (Q1 → Q3) were as follows: *m*/*z* 235.3 → 86.1 for lidocaine, *m*/*z* 265.3 → 176.1 for tetracaine, and *m*/*z* 245.4 → 96.1 for the internal standard (lidocaine-d10). The chromatographic separation was achieved on an HSS T3 C18 column (50 × 2.1 mm, 1.8 μm; Waters, Milford, MA, USA) at 45 °C, with a SIL20-AC autosampler (Shimadzu, Kyoto, Japan). The mobile phase consisted of ultrapure water with 10 mM ammonium acetate (eluent A) and acetonitrile (eluent B), which were applied in gradient mode at a flow rate of 0.3 mL/min.

The validation process was executed in accordance with the established guidelines set forth by the European Medicines Agency (EMA) and the Food and Drug Administration (FDA). The calibration curves were accepted if a minimum of 75% of the samples were within ±20% of the nominal values (±25% for the LLOQ), and if at least 66% of the quality control samples met these criteria. The instrumental data were acquired and processed with Analyst™ v1.4.2 (Sciex, USA).

### 2.6. Pharmacokinetic Analysis

Pharmacokinetic analysis was performed using non-compartmental methods adapted to intravenous and topical administration routes. The following parameters were determined: T_last_ (time of the last measurable plasma concentration), T_max_ (time to reach maximum concentration; topical only), C_0_ (extrapolated concentration at time 0; intravenous bolus only), C_max_ (maximum observed concentration; topical only), C_max/D_ (dose-normalized C_max_), AUC_last_ (area under the concentration–time curve from dosing to the last measurable concentration), AUC_last/D_ (dose-normalized AUC_last_), AUC_0–∞_ (area under the concentration–time curve from time 0 to infinity), AUC_0–∞/D_ (dose-normalized AUC_0–∞_), λ_z_ (elimination rate constant), t_½_ (elimination half-life), CL (total systemic clearance; intravenous only), V_z_ (volume of distribution during the elimination phase; intravenous only), V_ss_ (apparent volume of distribution at steady state; intravenous only), MRT (mean residence time; intravenous only), and F (absolute bioavailability). Where applicable, partial AUC values and their dose-normalized counterparts were also calculated. Absolute systemic bioavailability (F%) for topical administration was calculated using the area under the curve from time zero to the last measurable concentration (AUC_0-tlast_). This approach was selected to minimize extrapolation errors associated with the uncharacterized terminal phase observed in the topical arm (flip-flop kinetics) within the 24 h sampling window. Calculations involved normalizing for the dose difference between the formulations and the intravenous reference, ensuring a direct and valid comparison of formulation efficiency, regardless of differences in drug concentration.

All analyses were performed using Phoenix WinNonlin software (version 6.4, Certara, Radnor, PA, USA), which was used for both pharmacokinetic calculations and descriptive statistics within the toxicokinetic evaluation. Data from the intravenous arm were analyzed using a non-compartmental IV Bolus model, as the infusion duration (~1 min) was considered negligible relative to the total sampling time. Calculations (specifically non-compartmental analysis using the linear trapezoidal method for ascending concentrations and the log-linear trapezoidal method for descending concentrations) were conducted in accordance with the algorithms described by Gabrielsson et al. [38].

### 2.7. Correction of Tetracaine Concentrations Applied in Pharmacokinetic Calculations

In order to obtain reliable pharmacokinetic estimates for tetracaine, plasma concentrations were corrected for the compound’s instability in the biological matrix. During the course of analytical validation, tetracaine levels in minipig plasma were shown to decrease by 35.5% after 1.5 h of storage on ice. The aforementioned instability was the subject of a series of experiments, the results of which demonstrated progressive degradation in non-acidified plasma. However, these experiments also confirmed full stability in samples acidified with 1% formic acid. The detailed results of this validation are provided in the Supplementary Materials (Table S21).

In order to mitigate the effects of this phenomenon, plasma samples were acidified to a final concentration of 1% (*v*/*v*) formic acid prior to processing.

Since immediate acidification at the bedside was not feasible, a post hoc mathematical correction was applied based on ex vivo stability data generated during method validation. Stability tests confirmed a reproducible, time-dependent degradation of tetracaine in non-acidified plasma stored on ice (35.5% loss over 1.5 h) (Table S21 in Supplementary Materials). Correction factors, calculated as the inverse of the observed recovery rates (81.7% and 70.6%), were applied to reconstruct plasma concentrations: 1.22 (for 17–44 min delay) and 1.42 (for 45–65 min delay). While this approach introduces uncertainty, the linearity of degradation observed in validation samples supports the approximation for this exploratory dataset.

### 2.8. Statistical Analysis

The pharmacokinetic parameters were subsequently summarized using arithmetic means, standard deviations, and coefficients of variation. The accuracy and precision of the measurements were also assessed. Log-linear regression was applied to extrapolate C_0_ values subsequent to intravenous administration (based on the initial two time points if C_2_ < C_1_, otherwise C_1_ was used), to ascertain the terminal elimination rate constant λz (based on log-linear points of the terminal phase), and to calculate the elimination half-life t½ (using at least three time points with r^2^ ≥ 0.8).

For the purpose of determining the optimal dosage, reliable regression analysis was not feasible because the plasma concentration–time profiles lacked a distinct log-linear terminal phase. This phenomenon is indicative of flip-flop kinetics, wherein the rate of absorption from the skin serves as the rate-limiting step. This limitation did not impact the primary study endpoint, as absolute bioavailability (F%) was calculated based on AUC values.

All pharmacokinetic calculations, including non-compartmental analysis and descriptive statistics, were performed using Phoenix WinNonlin software (version 6.4, Certara, USA). The acquisition and processing of bioanalytical data were conducted using Analyst™ software (version 1.4.2, Sciex, USA).

Due to the exploratory nature of the study (*n* = 3), statistical power was inherently limited. Consequently, statistical analyses were conducted primarily to assess the directional consistency of observed trends across subjects rather than to provide confirmatory hypothesis testing. A Wilcoxon signed-rank test was employed to compare key pharmacokinetic parameters (F%, C_max_, AUC_last_) and skin concentrations between the Z4T4L4 and Pliaglis formulations. A *p*-value of less than 0.05 was considered statistically significant.

## 3. Results

The present study evaluated the pharmacokinetics and bioavailability of lidocaine hydrochloride and tetracaine hydrochloride following intravenous and topical administration in male Göttingen minipigs. The pharmacokinetic profiles were subsequently assessed following three distinct administration schemes: intravenous injection (Period 1), topical application of Pliaglis (Period 2), and topical application of Z4T4L4 (Period 3).

### 3.1. General Observations

It is noteworthy that no mortality occurred during the study. Following the administration of intravenous doses of lidocaine and tetracaine in Period 1, two of the three animals exhibited transient clinical signs, including mild lethargy, tremors, opisthotonosis, ventrolateral recumbency, blinking, moderate ataxia, and mild gait disturbances. The occurrence of these events was spontaneous. The local skin reactions observed in all animals following topical administration of Pliaglis (Period 2) and Z4T4L4 (Period 3) were characterized by mild to moderate erythema and scab formation. The reactions manifested in a localized manner, exhibited self-limiting properties, and did not impede the ongoing progression of the study. Initial body weight measurements ranged from 16 to 20 kg, with subsequent increases observed over the course of the study.

### 3.2. Bioanalysis

Individual and mean plasma concentration data are provided in the Supplementary Materials (Tables S1–S9).

The corrected tetracaine data following intravenous dosing (Period 1) are documented in Tables S1 and S2, while the lidocaine values are presented in Table S3. The corresponding data following the topical application of Pliaglis (Period 2) are presented in Tables S4–S6 and those following the administration of Z4T4L4 (Period 3) are in Tables S7–S9. The concentration of tetracaine and lidocaine in the skin is presented in Tables S10 and S11, respectively.

Following intravenous administration, the mean peak plasma concentrations of tetracaine and lidocaine were recorded. The mean peak plasma concentrations of tetracaine were approximately 150–160 ng/mL at 0.25 h and 850–900 ng/mL at 0.083 h. The mean peak plasma concentrations of lidocaine were recorded as 850–900 ng/mL at 0.083 h. Following topical administration of Pliaglis, the plasma concentrations ranged from the lower limit of quantification (LLOQ) to 7.90 ng/mL (tetracaine at 1 h) and from the LLOQ to 56.5 ng/mL (lidocaine at 2 h). For Z4T4L4, the range of values was from the lower limit of quantification (LLOQ) to 4.34 ng/mL of tetracaine at two hours (2 h) and from the LLOQ to 36.4 ng/mL of lidocaine at 2 h.

Furthermore, the topical application of the drug led to the detection of quantifiable levels of the drug in the skin tissue. Subsequent to the application of Pliaglis, the mean skin concentrations of tetracaine and lidocaine were recorded as 465 μg/g and 358 μg/g, respectively. Subsequent to Z4T4L4, concentrations were considerably lower, with an average of 46.1 μg/g for tetracaine and 33.6 μg/g for lidocaine, indicating a reduction greater than tenfold compared with Pliaglis. The concentration ranges for Pliaglis were 211–685 μg/g for tetracaine and 169–600 μg/g for lidocaine, while for Z4T4L4, the ranges were 34.0–67.0 μg/g and 19.8–53.6 μg/g, respectively.

These findings indicate that Pliaglis produced substantially higher local skin levels of both anesthetics compared with Z4T4L4.

### 3.3. Pharmacokinetics of Tetracaine

**Figure 1:**
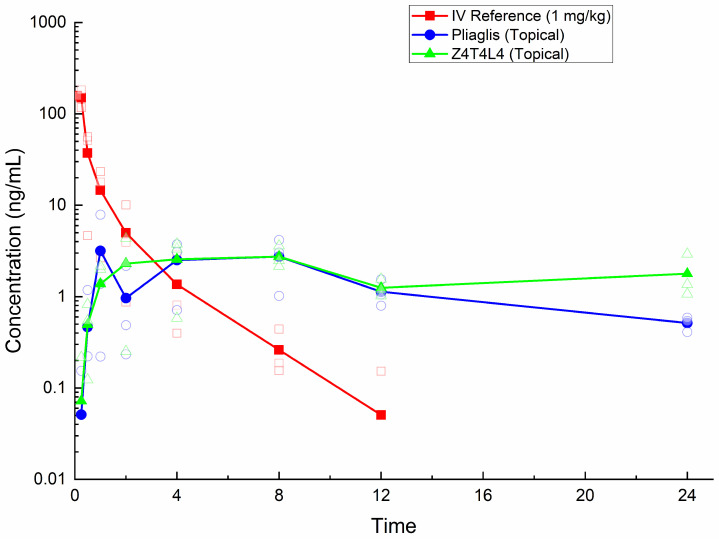
Mean and individual semi-logarithmic plasma concentration–time profiles of tetracaine after intravenous administration (1 mg/kg) and topical application of Pliaglis (10 g/animal) or Z4T4L4 (10 g/animal) in male Göttingen minipigs (*n* = 3). Solid lines with filled symbols represent arithmetic means, while small open symbols indicate individual data points. Concentrations below LLOQ were treated as zero. The semi-logarithmic scale highlights differences in absorption and elimination phases. Error bars were omitted in favor of individual data points to provide transparency regarding variability given the small sample size. Detailed individual data remain available in the Supplementary Materials.

The mean pharmacokinetic parameters of tetracaine, as determined by corrected plasma concentrations, are outlined in Table 3. The Supplementary Materials contain detailed individual and mean values, including standard deviation (SD) and coefficient of variation (CV%). Tables S12 and S13 detail the intravenous dosing, while Tables S15 and S16 provide information on Pliaglis and Tables S18 and S19 offer data on Z4T4L4. The mean plasma concentration–time profiles are presented in Figure 1.

Following the intravenous administration of tetracaine at a dosage of 1 mg/kg, the observed maximal plasma concentrations (C_max_) ranged from 82.4 to 129 ng/mL for animals with successful early sampling. The mean extrapolated concentration at time zero (C_0_) was determined to be 135 ng/mL, and the time of the last quantifiable concentration (T_last_) ranged from 8 to 12 h. The mean systemic exposure, expressed as AUC_0–∞_ was calculated to be 79.8 h·ng/mL. The mean clearance was found to be 19,900 mL/h/kg, indicating a high-clearance compound consistent with rapid hydrolysis by plasma esterases [15]. The volume of distribution at steady state (V_ss_) averaged 24,500 mL/kg, reflecting extensive tissue distribution. The mean terminal half-life was determined to be 1.85 h.

Subsequent to the topical application of Pliaglis (10 g/animal), the time to maximum concentration (T_max_) ranged from 1 to 8 h, with a mean C_max_ of 4.22 ng/mL and an area under the curve (AUC) of 34.6 h·ng/mL. The final sampling point occurred at the 24 h mark. The mean terminal half-life following topical administration was 6.37 h, which is longer than the mean terminal half-life following intravenous administration. This finding is consistent with flip-flop kinetics, in which the rate of absorption from the skin is slower than the rate of elimination in the systemic circulation [22]. The mean absolute bioavailability (F%) of tetracaine from Pliaglis was 1.26%.

In the subsequent analysis, the concentration of C_max_, following Z4T4L4, was found to be marginally lower (3.41 ng/mL), with T_max_ ranging between 2 and 8 h. The mean AUC_last_ value was determined to be 44.1 h·ng/mL, and the absolute bioavailability was calculated to be 3.34%, which is more than double that observed for Pliaglis. The presence of a terminal log-linear phase could not be ascertained, thereby impeding the calculation of the half-life. The enhanced systemic exposure observed with Z4T4L4, despite reduced skin concentrations, suggests that the formulation’s penetration-enhancing carrier facilitated more efficient systemic uptake.

### 3.4. Pharmacokinetics of Lidocaine

**Figure 2:**
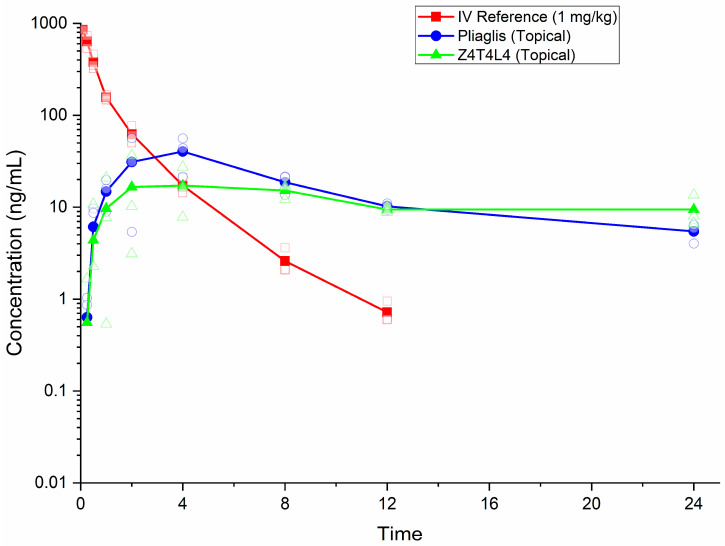
Mean and individual semi-logarithmic plasma concentration–time profiles of lidocaine after intravenous administration (1 mg/kg) and topical application of Pliaglis (10 g/animal) or Z4T4L4 (10 g/animal) in male Göttingen minipigs (*n* = 3). Solid lines with filled symbols represent arithmetic means, while small open symbols indicate individual data points. Concentrations below LLOQ were treated as zero. The semi-logarithmic scale highlights differences in absorption and elimination phases. Error bars were omitted in favor of individual data points to provide transparency regarding variability given the small sample size. Detailed individual data remain available in the Supplementary Materials.

The mean pharmacokinetic parameters of lidocaine are summarized in Table 4, and the corresponding mean plasma concentration–time profiles are presented in Figure 2. Detailed individual data are available in the Supplementary Materials (Tables S14, S17, and S20).

Following intravenous administration of 1 mg/kg, lidocaine reached a mean extrapolated C_0_ of 869 ng/mL. The last quantifiable concentration (T_last_) occurred at 12 h, and systemic exposure, expressed as AUC_0–∞_, averaged 681 h·ng/mL. The systemic clearance was 1490 mL/h/kg, indicating moderate clearance, while the steady-state distribution volume (V_ss_) was 1610 mL/kg, consistent with extensive tissue distribution. The mean terminal half-life was 1.62 h, which is consistent with the 1.5–2.0 h range reported in human studies [11,12]. This finding provides internal reference PK values in the Göttingen minipig necessary for calculating absolute systemic bioavailability within this experimental model.

The topical administration of the substance resulted in markedly lower systemic concentrations. Subsequent to the administration of Pliaglis, the mean concentration of C_max_ was recorded at 40.6 ng/mL, with a median time to maximum concentration (T_max_) ranging from 2 to 4 h. The area under the concentration–time curve (AUC_last_) was determined to be 371 h·ng/mL, and the apparent elimination half-life was calculated to be 9.58 h, which exceeded the duration observed following intravenous administration. This observation is indicative of flip–flop kinetics. The absolute bioavailability of the substance was determined to be 1.41%. Following the administration of Z4T4L4, the maximum observed concentration (C_max_) was found to be 23.1 ng/mL, with a time to reach maximum concentration (T_max_) ranging from 2 to 8 h. The area under the curve (AUC) averaged 278 h·ng/mL, and the terminal phase extended up to 24 h. The absence of a clearly defined terminal log-linear phase precluded reliable estimation of λ_z_ and t_½_. However, a trend towards higher systemic bioavailability was observed in comparison to Pliaglis (1.98%).

The modest increase in F% with Z4T4L4, despite lower skin concentrations, parallels the findings for tetracaine, suggesting formulation-driven enhancement of dermal penetration and systemic uptake. Lidocaine’s physicochemical properties, particularly its pKa (7.9) and moderate lipophilicity, render it sensitive to formulation pH and excipients, which may alter the unionized fraction and facilitate skin permeation [20].

### 3.5. A Potential Disconnect Between Skin Concentrations and Systemic Exposure

The topical administration of the substance in question resulted in a marked increase in the percentage of the substance found in the skin. Despite lower cutaneous concentrations measured at a single biopsy time point, Z4T4L4 yielded higher systemic exposure (AUC_0–tlast_) and F%, suggesting enhanced transdermal flux and/or altered depot kinetics rather than increased local retention. Following the administration of Pliaglis (Period 2), the mean drug levels in the skin were recorded as 465 μg/g for tetracaine and 358 μg/g for lidocaine. In contrast, Z4T4L4 (Period 3) resulted in approximately tenfold lower values, with tetracaine levels measuring 46.1 μg/g and lidocaine levels measuring 33.6 μg/g. Notwithstanding this substantial decrease in dermal deposition, systemic bioavailability exhibited a numerical tendency of being elevated with Z4T4L4.

Intravenous administration of 1 mg/kg of each agent confirmed the distinct pharmacokinetic behavior of both. The pharmacokinetic profile of tetracaine revealed high clearance (19,900 mL/h/kg), extensive distribution (V_ss_ = 24,500 mL/kg), and a brief terminal half-life (1.85 h). Lidocaine demonstrated moderate clearance (1490 mL/h/kg), extensive distribution (V_ss_ = 1610 mL/kg), and a half-life of 1.62 h, consistent with previously reported findings [11,12]. These pilot data support the utility of the Göttingen minipig as a within-study model for comparative assessment of dermal formulation performance; extrapolation to human systemic disposition should be made cautiously.

Following topical administration, the time to maximum plasma concentration (T_max_) ranged from 1 to 8 h for tetracaine and 2–8 h for lidocaine with Z4T4L4, compared to 1–8 h and 2–4 h with Pliaglis, respectively. For tetracaine, the mean absolute bioavailability was 1.26% for Pliaglis and 3.34% for Z4T4L4. For lidocaine, the values recorded were 1.41% and 1.98%, respectively. In both cases, systemic exposure was greater for Z4T4L4 despite lower dermal drug reservoirs. Furthermore, tetracaine exhibited an augmented apparent half-life following the topical application of Pliaglis (6.37 h), a hallmark of flip-flop kinetics, wherein the rate of absorption is less rapid compared to the rate of systemic elimination [22]. This observation does not contradict the known rapid systemic clearance of tetracaine (t_1/2_ = 1.85 h after IV administration, as confirmed in this study and reported in Table 3 and Table S13). Instead, it is a clear manifestation of “flip-flop kinetics,” where the apparent terminal half-life is governed by the rate-limiting dermal absorption process rather than the rapid systemic elimination.

A formal statistical comparison revealed a consistent numerical trend toward higher absolute bioavailability for Z4T4L4 (Wilcoxon signed-rank test, *p* = 0.1088). It should be noted that with a sample size of *n* = 3, the statistical power is mathematically constrained, and even a consistent unidirectional effect across all subjects may not yield a *p*-value below the traditional 0.05 threshold in non-parametric testing. Therefore, the observed *p*-value reflects a consistent trend rather than an absence of effect, warranting verification in a larger cohort. Furthermore, no statistically significant differences were identified for C_max_ between the formulations for either tetracaine (*p* = 1.0) or lidocaine (*p* = 0.1088). In contrast, while mean drug concentrations in the skin were numerically over tenfold higher after Pliaglis application compared to Z4T4L4 for both tetracaine (465 vs. 46.1 μg/g) and lidocaine (358 vs. 33.6 μg/g), these large differences also did not reach statistical significance (*p* = 0.1088 for both). This result is likely due to the study’s limited statistical power.

## 4. Discussion

The present pilot study provided a comparative evaluation of the pharmacokinetics of lidocaine and tetracaine following intravenous and topical administration in male Göttingen minipigs. The within-subject, fixed-sequence design helped minimize inter-individual variability by allowing each animal to serve as its own reference across periods.

It is imperative to underscore that this exploratory pilot study was deliberately designed with a sample size of three animals, in accordance with the 3Rs principles. Therefore, all comparisons between formulations are based on numerical differences in mean pharmacokinetic parameters. As a result, these comparisons should be interpreted as descriptive trends rather than statistically significant outcomes. Despite the implementation of an exploratory Wilcoxon signed-rank test, the resulting differences were not statistically significant (*p* = 0.1088 for both drugs). As noted in the results, this value reflects the mathematical constraint of non-parametric testing with *n* = 3 rather than an absence of effect, indicating a consistent unidirectional trend. This absence of statistical significance, attributable to the study’s limited statistical power, hinders the ability to draw definitive conclusions regarding the superiority of either formulation. Consequently, the observed numerical trends should be regarded as preliminary and hypothesis-generating. Moreover, the notable inter-individual variability observed for certain parameters, as indicated by the standard deviations in Table 3 and Table 4, further underscores the preliminary nature of these findings.

Therefore, the observations discussed below require confirmation in subsequent, adequately powered studies before definitive conclusions can be drawn.

Initially, the systemic disposition of both compounds following intravenous administration was consistent with the previously described pharmacokinetic characteristics. The pharmacokinetic profile of tetracaine was characterized by its high clearance (19,900 mL/h/kg), substantial apparent distribution volume (V_ss_ = 24,500 mL/kg), and brief half-life (1.85 h). These observations are indicative of its rapid hydrolysis by plasma esterases [15]. Lidocaine demonstrated moderate clearance (1490 mL/h/kg), extensive tissue distribution (V_ss_ = 1610 mL/kg), and a terminal half-life (1.62 h) within the range reported in humans [11,12]. These intravenous data provided the necessary reference clearance values to calculate absolute bioavailability (F_abs_) within the current experimental model. While the Göttingen minipig is established as a premier model for cutaneous permeation, we acknowledge that interspecies scaling of systemic disposition typically relies on multi-species allometry (e.g., rat, dog, monkey) [39]. Therefore, the IV data presented here primarily serve to validate the internal consistency of the bioavailability estimation rather than to predict human systemic pharmacokinetics directly.

Secondly, the topical application of the substance revealed a paradoxical relationship between skin deposition and systemic uptake. The reference product Pliaglis, containing 7%/7% lidocaine/tetracaine, produced substantially higher dermal concentrations—over tenfold higher—than Z4T4L4 (4%/4%) (mean skin concentrations for Pliaglis were 358 μg/g for lidocaine and 465 μg/g for tetracaine, compared to 33.6 μg/g and 46.1 μg/g for Z4T4L4, respectively). Notwithstanding this observation, Pliaglis exhibited diminished absolute bioavailability for both anesthetics. Specifically, after normalizing for the applied dose, the bioavailability (F%) of tetracaine from Z4T4L4 was more than double that of Pliaglis (3.34% vs. 1.26%), and the bioavailability of lidocaine was also higher (1.98% vs. 1.41%). This finding indicates that the novel Z4T4L4 carrier system facilitates the delivery of active compounds to the systemic circulation with greater efficiency per unit of dose compared to the reference product. While limited by a single sampling time point (90 min), the data suggest that the magnitude of the cutaneous drug reservoir at the end of the dosing period may not be linearly predictive of the total systemic exposure (AUC). This implies that the Z4T4L4 carrier system may facilitate rapid transdermal flux, minimizing skin retention compared to the reference product. Instead, formulation characteristics, particularly carrier composition and physicochemical properties, appear to critically influence transdermal flux [19,22,40]. These divergent pharmacokinetic profiles suggest distinct permeation mechanisms driven by the vehicle properties. The reference product (Pliaglis), a eutectic mixture in a viscous base, appears to favor the formation of an intracutaneous depot, potentially facilitated by follicular accumulation [41], which explains the higher dermal retention despite lower systemic bioavailability. In contrast, the Z4T4L4 hydro-alcoholic carrier is designed to maximize the thermodynamic activity of the active ingredients [42], thereby promoting rapid transepidermal flux directly into the systemic circulation rather than superficial deposition. This ‘flux-dominant’ mechanism likely contributes to the distinct late-phase release kinetics observed.

The elevated F% recorded for Z4T4L4 (3.34% for tetracaine and 1.98% for lidocaine), despite diminished skin levels, might be attributable to the presence of penetration-enhancing excipients. These excipients facilitate the partitioning of the unionized drug fraction into more profound skin layers, thereby enhancing systemic transfer. Reduced binding or retention in superficial compartments could further improve penetration toward the dermal vasculature and enhance systemic absorption [19,40]. A similar phenomenon has been observed in other topical anesthetic systems, where enhanced systemic uptake did not correlate with maximum dermal deposition [20].

These preliminary findings may have clinical implications. First, the drug strength indicated on the label does not reliably predict systemic exposure. This is because lower-strength formulations may still achieve comparable or greater bioavailability if equipped with effective carriers. It is important to clarify that while systemic exposure is not the therapeutic goal for local anesthetics, in the context of this formulation development study, it serves as a kinetic indicator of the carrier’s ability to overcome the stratum corneum barrier. The observed enhancement in systemic bioavailability (F_abs_) for Z4T4L4, despite lower cutaneous retention, suggests a ‘flux-dominant’ permeation profile distinct from the reference product’s ‘depot-forming’ behavior. Secondly, while the peak plasma concentrations observed in this pilot study (C_max_ = 23.1 ng/mL) remain negligible compared to plasma levels associated with systemic toxicity in clinical practice (reported as >5000 ng/mL [43]), the mechanism of augmented systemic absorption may engender an elevated risk of adverse events in clinical scenarios involving larger application surface areas or repeated dosing. Therefore, the benefit-risk profile must be carefully evaluated in future dose-finding studies. Consequently, the formulation design must meticulously balance local efficacy with systemic safety.

The present findings complement earlier pharmacokinetic studies of topical anesthetics. While the impact of Critical Quality Attributes (CQAs) on cutaneous bioavailability has been extensively documented for anti-infectives like acyclovir [44], comparative in vivo data linking specific vehicle mechanisms to systemic safety for anesthetic combinations remain limited. Previous studies often evaluated lidocaine or tetracaine separately or employed heterogeneous designs, precluding direct comparisons [31,32,33]. It is important to note that this pilot study is among the first exploratory comparisons of Pliaglis with a penetration-enhancing alternative under identical experimental conditions. These findings underscore the critical observation that systemic exposure is formulation-dependent.

Several limitations should be considered. The study was designed as an exploratory pilot constrained to three animals (*n* = 3) in strict adherence to the 3Rs principle (Reduction) to minimize animal use in preliminary investigations. We acknowledge that this limited sample size contributed to the high relative standard deviations (RSD > 50%) observed for primary pharmacokinetic parameters, reflecting the inherent inter-individual variability of dermal absorption in the minipig model. Consequently, the study was not powered to detect statistical significance, and the observed differences should be interpreted as descriptive trends. Furthermore, the notation “insufficient data” (ID) in the summary tables reflects the mathematical constraint of non-compartmental analysis where the terminal elimination phase could not be reliably defined (r^2^ < 0.8) within the 24 h sampling window due to flip-flop kinetics [45], rather than missing bioanalytical data. Although the 24 h sampling scheme captured the absorption phase and peak exposure (C_max_), it did not permit comprehensive characterization of terminal elimination following topical administration. Consequently, the reliance on AUC_0-tlast_ (due to the limitation in defining λ_z_ for extrapolation) implies that the reported absolute bioavailability values (F_abs_) may underestimate the total systemic exposure, particularly given the slow release kinetics typical of dermal depots. Additionally, the bioanalytical scope of this pilot study was restricted to the parent compounds (lidocaine and tetracaine) to quantify the comparative absorption rates of the formulations. Active metabolites, such as monoethylglycinexylidide (MEGX) for lidocaine, were not quantified, although their assessment is planned for future definitive studies to fully elucidate the systemic disposition. Regarding the removal of the formulation, although care was taken to avoid organic solvents that could alter permeability (e.g., alcohol-induced solubilization of crystalline API), the residual amount of formulation removed after 60 min was not quantified. Moreover, because antisepsis before biopsy included alcohol, residual drug in the most superficial strata could have been reduced, potentially biasing the single time point skin concentration snapshot; however, the same procedure was applied across periods, preserving the validity of between-formulation comparisons. Consequently, the bioavailability estimates are based on the nominal applied dose rather than the actual absorbed dose (mass balance), which may result in an underestimation of the ‘absorbed fraction’ efficiency but accurately reflects the systemic exposure relative to the clinical application dose. Furthermore, the presence of residual variability in skin physiology may potentially contribute to the influence of absorption kinetics, the within-subject, fixed-sequence design. Furthermore, while Göttingen minipig skin is morphologically similar to human skin [46], species-specific differences in follicular density, stratum corneum thickness, and cutaneous blood flow may influence absolute absorption rates [37]. Therefore, the reported bioavailability values should be interpreted as comparative formulation performance rather than direct predictors of human clinical exposure. In future studies, it is imperative to extend the sampling period (e.g., to 48–72 h) and employ a re-validated bioanalytical method with increased sensitivity (lower LLOQ) to fully characterize the terminal elimination phase, increase the sample size, and incorporate advanced cutaneous sampling techniques such as Dermal Open Flow Microperfusion (dOFM) or spatial biopsy mapping to resolve local distribution kinetics. Additionally, the intravenous pharmacokinetic parameters established in this study will serve as foundational input for future Physiologically Based Pharmacokinetic (PBPK) modeling to describe the dermal absorption process and predict human clinical exposure. These additional measures will facilitate a more comprehensive elucidation of the intricacies underlying penetration.

In summary, the pilot data demonstrate that the systemic bioavailability of lidocaine and tetracaine is strongly formulation-dependent and not linearly correlated with dermal concentrations. These results may inform future efforts toward the development of optimized penetration-enhancing carriers that may reduce active drug content while preserving or improving therapeutic efficacy. In order to ensure the safe and effective use of novel topical anesthetic products, it is imperative that the clinical validation of this bioavailability paradox be conducted.

The interpretation of tetracaine pharmacokinetics is constrained by its rapid hydrolysis in plasma. Although correction factors were derived from validated stability profiles, the reconstruction of plasma concentrations represents an estimation rather than direct measurement. Additionally, the use of a single biopsy time point precludes the calculation of cutaneous pharmacokinetic parameters (such as skin AUC), limiting the comparison to a ‘snapshot’ of skin retention rather than total tissue exposure.

## 5. Conclusions

These pilot data suggest that the pharmacokinetic behavior of lidocaine and tetracaine may be highly dependent on the formulation applied topically. In this study, the reference product Pliaglis, despite producing substantially higher drug concentrations in skin tissue, was observed to have lower absolute systemic bioavailability compared with the novel Z4T4L4 formulation.

In contrast, Z4T4L4, which contained a lower drug content, exhibited a tendency to elicit higher F% values for both anesthetics, underscoring a plausible function of carrier systems in modulating dermal absorption and systemic uptake.

These preliminary findings from the present pilot study suggest a potential bioavailability paradox: local skin concentration may not directly predict systemic exposure. The results underscore the potential significance of formulation design in achieving a balance between therapeutic efficacy and systemic safety. The findings provide a rationale for further investigations and clinical translation, which must be validated in studies with sufficient statistical power to confirm this apparent paradox.

## 6. Patents

The Z4T4L4 formulation described in this study is covered by patents cited in the manuscript, including proprietary composition and manufacturing process claims held by Biotts SA.

## Figures and Tables

**Table 1:** Experimental design and dosing regimen.

Period ^b^	Test Item Id.	Dose Level (mg/kg/Test Item)	Dose Volume ^a^ (mL/kg)	Dose Concentration (mg/mL)	Dose Route	Number of Animals Per Period	Animal IDs
						Males	Males
1	Lidocaine (HCl) and Tetracaine (HCl)	1 ^d^	0.5	2	Intravenous	3	1–3
**Period ^b^**	**Test Item Id.**	**Dose Concentration (%/Test Item)**	**Treatment Area (cm^2^)**	**Dose Amount**	**Dose Route**	**Number of Animals Per Period**	**Animal IDs**
2	Pliaglis	7	100	10 g/100 cm^2^	Topical	3	1–3
3	Z4T4L4	4	100	10 g/100 cm^2^			

The study was conducted using a fixed-sequence, within-subject, three-period design (*n* = 3). The ‘Animal IDs’ column refers to the unique identification numbers of these animals. Id. = identification. ^a^ Based on the most recent body weight measurement; ^b^ There was a wash-out period of one week between Periods 1 and 2 and of two weeks between Periods 2 and 3; Animals were treated for 60 min; ^d^ correction factor of 1.23 for lidocaine (HCL) and 1.14 for tetracaine (HCL) was used.

**Table 2:** Bioanalytical sample collection schedule.

	Time Postdose (Period 1) and Time After Start of Application (Periods 2 and 3)								
Period Numbers	5 min (0.083 h)	15 min (0.25 h)	30 min (0.5 h)	1 h	2 h	4 h	8 h	12 h	24 h
1	X	X	X	X	X	X	X	X	X
2		X	X	X	X	X	X	X	X
3		X	X	X	X	X	X	X	X

X = Sample collected.

**Table 3:** Mean pharmacokinetic parameters of tetracaine (based on corrected plasma concentrations).

Parameters		1 mg/kg iv. (Period 1)	10 g/Animal Pliaglis Topical (Period 2)	10 g/Animal Z4T4L4 Topical (Period 3)
T_last_	h	8–12 ^a^	24	24
C_0_	ng/mL	135 ± 121	-	-
T_max_	h	-	1–8 ^a^	2–8 ^a^
C_max_	ng/mL	107.8 ± 80.60 ^b^	4.22 ±3.46	3.41 ± 1.14
AUC_last_	h∙ng/mL	79.3 ± 48.10	34.6 ± 16.20	44.1 ± 16.20
AUC_last_/Dose	h∙kg∙ng/mL/mg	79.3 ± 48.10	0.919 ± 0.46	2.15 ± 0.85
AUC_0–∞_	h∙ng/mL	79.8 ± 48.00	48.5 ± ID	n/a
AUC_0–∞_/Dose	h∙kg∙ng/mL/mg	79.8 ± 48.00	1.32 ± ID	n/a
λ_z_	1/h	0.395 ± 0.11	0.114 ± ID	n/a
T_1/2_	h	1.85 ± 0.55	6.37 ± ID	n/a
Cl	mL/h/kg	19,900 ± 18,300	-	-
V_z_	mL/kg	62,600 ± 72,700	-	-
V_ss_	mL/kg	24,500 ± 29,800	-	-
MRT	h	1.01 ± 0.42	-	-
F	%	-	1.26 ± 0.27	3.34 ± 1.56

Note: Data are presented as mean ± SD unless otherwise indicated. -/n/a: not applicable; ID: insufficient data; ^a^: range; /Dose: dose-normalized to 1 mg/kg; ^b^: Observed maximal concentration (C_max_obs_) derived from the first quantifiable sample or the peak of the early distribution phase, distinct from the extrapolated C_0_.

**Table 4:** Mean Pharmacokinetic Parameters of Lidocaine.

Parameters		1 mg/kg iv. (Period 1)	10 g/Animal Pliaglis Topical (Period 2)	10 g/Animal Z4T4L4 Topical (Period 3)
T_last_	h	12	24	24
C_0_	ng/mL	869 ± 67.40	-	-
T_max_	h	-	2–4 ^a^	2–8 ^a^
C_max_	ng/mL	-	40.6 ± 17.80	23.1 ± 11.60
AUC_last_	h∙ng/mL	679 ± 97.30	371 ± 99.20	278 ± 61.30
AUC_last_/Dose	h∙kg∙ng/mL/mg	679 ±97.30	9.78 ± 3.69	13.7 ± 4.69
AUC_0–∞_	h∙ng/mL	681 ± 98.00	418 ± ID	n/a
AUC_0–∞_/Dose	h∙kg∙ng/mL/mg	681 ± 98.00	11.7 ± ID	n/a
λ_z_	1/h	0.429 ± 0.04	0.0825 ± ID	n/a
T_1/2_	h	1.62 ± 0.15	9.58 ± ID	n/a
Cl	mL/h/kg	1490 ± 198	-	-
V_z_	mL/kg	3460 ± 204	-	-
V_ss_	mL/kg	1610 ± 139	-	-
MRT	h	1.08 ± 0.07	-	-
F	%	-	1.41 ± 0.34	1.98 ± 0.38

Note: Data are presented as mean ± SD unless otherwise indicated. -/n/a: not applicable; ID: insufficient data; ^a^: range; /Dose: dose-normalized to 1 mg/kg.

## Data Availability

All data supporting the reported results are contained within the manuscript and its Supplementary Materials. Data related to the Z4T4L4 formulation are available in the patent sources cited in the publication.

## Supplementary Materials

The following supporting information can be downloaded at https://www.mdpi.com/article/10.3390/pharmaceutics18010040/s1: Table S1: Bioanalysis Data of Tetracaine in Plasma after Single Intravenous Administration of 1 mg/kg Lidocaine HCl and Tetracaine HCl (Period 1); Table S2: Bioanalysis Data of Corrected Tetracaine in Plasma after Single Intravenous Administration of 1 mg/kg Lidocaine HCl and Tetracaine HCl (Period 1); Table S3: Bioanalysis Data of Lidocaine in Plasma after Single Intravenous Administration of 1 mg/kg Lidocaine HCl and Tetracaine HCl (Period 1); Table S4: Bioanalysis Data of Tetracaine in Plasma after Single Topical Administration of 10 g/animal Pliaglis (Period 2); Table S5: Bioanalysis Data of Corrected Tetracaine in Plasma after Single Topical Administration of 10 g/animal Pliaglis (Period 2); Table S6: Bioanalysis Data of Lidocaine in Plasma after Single Topical Administration of 10 g/animal Pliaglis (Period 2); Table S7: Bioanalysis Data of Tetracaine in Plasma after Single Topical Administration of 10 g/animal Z4T4L4 (Period 3); Table S8: Bioanalysis Data of Corrected Tetracaine in Plasma after Single Topical Administration of 10 g/animal Z4T4L4 (Period 3); Table S9: Bioanalysis Data of Lidocaine in Plasma after Single Topical Administration of 10 g/animal Z4T4L4 (Period 3); Table S10: Bioanalysis Data of Tetracaine in Skin after Single Topical Administration of 10 g/animal; Table S11: Bioanalysis Data of Lidocaine in Skin after Single Topical Administration of 10 g/animal; Table S12: Pharmacokinetic Parameters for Tetracaine after Single Intravenous Administration of 1 mg/kg Lidocaine HCl and Tetracaine HCl (Period 1); Table S13: Pharmacokinetic Parameters for Corrected Tetracaine after Single Intravenous Administration of 1 mg/kg Lidocaine HCl and Tetracaine HCl (Period 1); Table S14: Pharmacokinetic Parameters for Lidocaine after Single Intravenous Administration of 1 mg/kg Lidocaine HCl and Tetracaine HCl (Period 1); Table S15: Pharmacokinetic Parameters for Tetracaine after Single Topical Administration of 10 g/animal Pliaglis (Period 2); Table S16: Pharmacokinetic Parameters for Corrected Tetracaine after Single Topical Administration of 10 g/animal Pliaglis (Period 2); Table S17: Pharmacokinetic Parameters for Lidocaine after Single Topical Administration of 10 g/animal Pliaglis (Period 2); Table S18: Pharmacokinetic Parameters for Tetracaine after Single Topical Administration of 10 g/animal Z4T4L4 (Period 3); Table S19: Pharmacokinetic Parameters for Corrected Tetracaine after Single Topical Administration of 10 g/animal Z4T4L4 (Period 3); Table S20: Pharmacokinetic Parameters for Lidocaine after Single Topical Administration of 10 g/animal Z4T4L4 (Period 3); Table S21: Stability of Tetracaine in Göttingen Minipig Plasma with and without Acidification: Peak Areas, Calculated Concentrations, and Accuracy over 0–1.5 Hours at 0–4 °C.
