# Supplementary material for: Dermal Concentration Versus Systemic Bioavailability of Topical Lidocaine and Tetracaine: An Exploratory Pharmacokinetic Pilot Study in Göttingen Minipigs
Source: Pharmaceutics. 2025 Dec 28;18(1):40. doi: 10.3390/pharmaceutics18010040 (PMC12845211; doi:10.3390/pharmaceutics18010040)
Supplement: Supplementary file 1 [file pharmaceutics-18-00040-s001.zip › pharmaceutics-4033237-supplementary.pdf]

Supplementary Materials

Dermal Concentration Versus Systemic Bioavailability of Topical Lidocaine and Tetracaine: An Exploratory Pharmacokinetic Pilot Study in Göttingen Minipigs

Paweł Biernat [1,2], Dawid Bursy [1,2], Dominik Marciniak [1,2], Konrad Krajewski [2,3], Jan Meler [1,2] and Radosław Balwierz [2,4,*]

[1] Department of Drug Forms Technology, Faculty of Pharmacy, Wrocław Medical University, 211 Borowska St., 50-556 Wroclaw, Poland
[2] Biotts SA, Wroclawska 44c St., Bielany Wroclawskie, 55-040 Wroclaw, Poland
[3] Faculty of Computer Science and Management, Wroclaw University of Science and Technology, 50-370 Wroclaw, Poland
[4] Institute of Chemistry, University of Opole, Oleska 48 St.,45-052 Opole, Poland
* Correspondence: radoslaw.balwierz@uni.opole.pl; Tel.: +48-77-452-71-13

Table of Contents

Table S1. Bioanalysis Data of Tetracaine in Plasma after Single Intravenous Administration of 1 mg/kg Lidocaine HCl and Tetracaine HCl (Period 1)............3
Table S2. Bioanalysis Data of Corrected Tetracaine in Plasma after Single Intravenous Administration of 1 mg/kg Lidocaine HCl and Tetracaine HCl (Period 1) 3
Table S3. Bioanalysis Data of Lidocaine in Plasma after Single Intravenous Administration of 1 mg/kg Lidocaine HCl and Tetracaine HCl (Period 1)............3
Table S4. Bioanalysis Data of Tetracaine in Plasma after Single Topical Administration of 10 g/animal Pliaglis (Period 2)............4
Table S5. Bioanalysis Data of Corrected Tetracaine in Plasma after Single Topical Administration of 10 g/animal Pliaglis (Period 2)............4
Table S6. Bioanalysis Data of Lidocaine in Plasma after Single Topical Administration of 10 g/animal Pliaglis (Period 2)............4
Table S7. Bioanalysis Data of Tetracaine in Plasma after Single Topical Administration of 10 g/animal Z4T4L4 (Period 3)............5
Table S8. Bioanalysis Data of Corrected Tetracaine in Plasma after Single Topical Administration of 10 g/animal Z4T4L4 (Period 3)............5
Table S9. Bioanalysis Data of Lidocaine in Plasma after Single Topical Administration of 10 g/animal Z4T4L4 (Period 3)............5
Table S10. Bioanalysis Data of Tetracaine in Skin after Single Topical Administration of 10 g/animal 6
Table S11. Bioanalysis Data of Lidocaine in Skin after Single Topical Administration of 10 g/animal 6
Table S12. Pharmacokinetic Parameters for Tetracaine after Single Intravenous Administration of 1 mg/kg Lidocaine HCl and Tetracaine HCl (Period 1)............6
Table S13. Pharmacokinetic Parameters for Corrected Tetracaine after Single Intravenous Administration of 1 mg/kg Lidocaine HCl and Tetracaine HCl (Period 1) 7
Table S14. Pharmacokinetic Parameters for Lidocaine after Single Intravenous Administration of 1 mg/kg Lidocaine HCl and Tetracaine HCl (Period 1)............7
Table S15. Pharmacokinetic Parameters for Tetracaine after Single Topical Administration of 10 g/animal Pliaglis (Period 2)............8
Table S16. Pharmacokinetic Parameters for Corrected Tetracaine after Single Topical Administration of 10 g/animal Pliaglis (Period 2)............8

Table S17. Pharmacokinetic Parameters for Lidocaine after Single Topical Administration of 10 g/animal Pliaglis (Period 2) ... 9
Table S18. Pharmacokinetic Parameters for Tetracaine after Single Topical Administration of 10 g/animal Z4T4L4 (Period 3) ... 9
Table S19. Pharmacokinetic Parameters for Corrected Tetracaine after Single Topical Administration of 10 g/animal Z4T4L4 (Period 3) ... 10
Table S20. Pharmacokinetic Parameters for Lidocaine after Single Topical Administration of 10 g/animal Z4T4L4 (Period 3) ... 10
Table S21. Stability of Tetracaine in Göttingen Minipig Plasma with and without Acidification: Peak Areas, Calculated Concentrations, and Accuracy over 0–1.5 Hours at 0–4 °C ... 11

Table S1. Bioanalysis Data of Tetracaine in Plasma after Single Intravenous Administration of 1 mg/kg Lidocaine HCl and Tetracaine HCl (Period 1)

Animal number	Time (h)								
	**0.083**	**0.25**	**0.5**	**1**	**2**	**4**	**8**	**12**	**24**
	Concentration (ng/mL)								
1	112	129	41.7	12.6	3.21	0.57	0.153	< LLOQ[1]	< LLOQ[1]
2	n/s	82.4	45.8	1.88	8.29	2.04	0.360	0.125	< LLOQ[1]
3	n/s	n/s	3.79	19.1	0.712	0.325	0.127	< LLOQ[1]	< LLOQ[1]
Mean	112	106	30.4	11.2	4.07	0.977	0.213	0.0415	0.00
SD	n/a	ID	23.2	8.70	3.86	0.925	0.128	0.0719	n/a
CV%	n/a	ID	76	78	95	95	60	173	n/a

LLOQ: below lower limit of quantification of 0.1 ng/mL; n/s: no sample taken; n/a: not applicable;
ID: insufficient data;
[1]: value set to 0 for descriptive statistics and pharmacokinetic evaluation.

Table S2. Bioanalysis Data of Corrected Tetracaine in Plasma after Single Intravenous Administration of 1 mg/kg Lidocaine HCl and Tetracaine HCl (Period 1)

Animal number	Time (h)								
	**0.083**	**0.25**	**0.5**	**1**	**2**	**4**	**8**	**12**	**24**
	Concentration (ng/mL)								
1	158	183	51.0	17.9	3.93	0.807	0.187	< LLOQ[1]	< LLOQ[1]
2	n/s	117	56.0	2.66	10.1	2.88	0.441	0.153	< LLOQ[1]
3	n/s	n/s	4.64	23.4	0.872	0.398	0.156	< LLOQ[1]	< LLOQ[1]
Mean	158	150	37.2	14.6	4.98	1.36	0.261	0.0508	0.00
SD	n/a	ID	28.3	10.7	4.72	1.33	0.156	0.0881	n/a
CV%	n/a	ID	76	73	95	98	60	173	n/a

LLOQ: below lower limit of quantification of 0.1 ng/mL; n/s: no sample taken; n/a: not applicable;
ID: insufficient data;
[1]: value set to 0 for descriptive statistics and pharmacokinetic evaluation.

Table S3. Bioanalysis Data of Lidocaine in Plasma after Single Intravenous Administration of 1 mg/kg Lidocaine HCl and Tetracaine HCl (Period 1)

Animal number	Time (h)								
	**0.083**	**0.25**	**0.5**	**1**	**2**	**4**	**8**	**12**	**24**
	Concentration (ng/mL)								
1	852	736	462	167	77.0	20.9	3.62	0.951	< LLOQ[1]
2	n/s	538	321	147	60.8	16.5	2.08	0.607	< LLOQ[1]
3	n/s	n/s	351	155	50.2	14.3	2.08	0.597	< LLOQ[1]
Mean	852	637	378	157	62.7	17.2	2.60	0.718	0.00
SD	n/a	ID	74.2	10.1	13.5	3.40	0.890	0.201	n/a
CV%	n/a	ID	20	6	22	20	34	28	n/a

LLOQ: below lower limit of quantification of 0.5 ng/mL; n/s: no sample taken; n/a: not applicable;
ID: insufficient data;
[1]: value set to 0 for descriptive statistics and pharmacokinetic evaluation.

Table S4. Bioanalysis Data of Tetracaine in Plasma after Single Topical Administration of 10 g/animal Pliaglis (Period 2)

Animal number	Time (h)							
	0.25	0.5	1	2	4	8	12	24
	Concentration (ng/mL)							
1	0.109	0.830	1.13	1.77	3.04	2.49	0.863	0.481
2	< LLOQ[1]	< LLOQ[1]	6.45	0.399	2.52	3.41	1.26	0.335
3	< LLOQ[1]	0.182	0.181	0.191	0.582	0.835	0.651	0.443
Mean	0.0362	0.337	2.59	0.787	2.05	2.24	0.924	0.42
SD	0.0626	0.436	3.38	0.858	1.30	1.30	0.308	0.076
CV%	173	129	131	109	63	58	33	18

LLOQ: below lower limit of quantification of 0.1 ng/mL;
[1]: value set to 0 for descriptive statistics and pharmacokinetic evaluation.

Table S5. Bioanalysis Data of Corrected Tetracaine in Plasma after Single Topical Administration of 10 g/animal Pliaglis (Period 2)

Animal number	Time (h)							
	0.25	0.5	1	2	4	8	12	24
	Concentration (ng/mL)							
1	0.154	1.18	1.38	2.17	3.73	3.04	1.06	0.589
2	< LLOQ[1]	< LLOQ[1]	7.90	0.488	3.08	4.17	1.54	0.41
3	< LLOQ[1]	0.222	0.221	0.234	0.712	1.02	0.797	0.542
Mean	0.0512	0.466	3.17	0.963	2.51	2.74	1.13	0.514
SD	0.0887	0.625	4.14	1.05	1.59	1.59	0.377	0.0931
CV%	173	134	131	109	63	58	33	18

LLOQ: below lower limit of quantification of 0.1 ng/mL;
[1]: value set to 0 for descriptive statistics and pharmacokinetic evaluation.

Table S6. Bioanalysis Data of Lidocaine in Plasma after Single Topical Administration of 10 g/animal Pliaglis (Period 2)

Animal number	Time (h)							
	0.25	0.5	1	2	4	8	12	24
	Concentration (ng/mL)							
1	1.03	9.68	19.7	56.5	56.1	21.5	9.36	5.91
2	< LLOQ[1]	< LLOQ[1]	8.93	5.40	21.4	21.2	10.2	4.03
3	0.867	8.68	15.9	31.1	43.7	13.6	11.0	6.42
Mean	0.633	6.12	14.8	31.0	40.4	18.7	10.2	5.45
SD	0.554	5.32	5.46	25.6	17.6	4.49	0.841	1.26
CV%	88	87	37	82	44	24	8	23

LLOQ: below lower limit of quantification of 0.5 ng/mL;
[1]: value set to 0 for descriptive statistics and pharmacokinetic evaluation.

Table S7. Bioanalysis Data of Tetracaine in Plasma after Single Topical Administration of 10 g/animal Z4T4L4 (Period 3)

Animal number	Time (h)							
	0.25	0.5	1	2	4	8	12	24
	Concentration (ng/mL)							
1	0.177	0.671	1.63	3.55	2.72	2.04	0.836	0.758
2	< LLOQ[1]	0.443	1.76	1.89	3.07	2.94	1.27	2.06
3	< LLOQ[1]	0.101	< LLOQ[1]	0.206	0.474	1.75	0.972	0.961
Mean	0.0589	0.405	1.13	1.88	2.09	2.24	1.02	1.26
SD	0.102	0.287	0.981	1.67	1.41	0.618	0.220	0.700
CV%	173	71	87	89	67	28	21	56

LLOQ: below lower limit of quantification of 0.1 ng/mL;
[1]: value set to 0 for descriptive statistics and pharmacokinetic evaluation.

Table S8. Bioanalysis Data of Corrected Tetracaine in Plasma after Single Topical Administration of 10 g/animal Z4T4L4 (Period 3)

Animal number	Time (h)							
	0.25	0.5	1	2	4	8	12	24
	Concentration (ng/mL)							
1	0.216	0.821	1.99	4.34	3.33	2.49	1.02	1.07
2	< LLOQ[1]	0.542	2.16	2.31	3.76	3.59	1.55	2.92
3	< LLOQ[1]	0.123	< LLOQ[1]	0.252	0.58	2.15	1.19	1.36
Mean	0.0721	0.495	1.38	2.30	2.56	2.74	1.25	1.78
SD	0.125	0.351	1.20	2.04	1.72	0.756	0.269	0.992
CV%	173	71	87	89	67	28	21	56

LLOQ: below lower limit of quantification of 0.1 ng/mL;
[1]: value set to 0 for descriptive statistics and pharmacokinetic evaluation.

Table S9. Bioanalysis Data of Lidocaine in Plasma after Single Topical Administration of 10 g/animal Z4T4L4 (Period 3)

Animal number	Time (h)							
	0.25	0.5	1	2	4	8	12	24
	Concentration (ng/mL)							
1	1.66	10.8	20.8	36.4	27.4	17.1	9.21	8.04
2	< LLOQ[1]	2.26	7.71	10.2	16.3	12.1	8.82	6.70
3	< LLOQ[1]	< LLOQ[1]	0.536	3.11	7.79	16.5	10.2	13.6
Mean	0.555	4.37	9.67	16.6	17.2	15.2	9.42	9.44
SD	0.961	5.72	10.3	17.6	9.83	2.72	0.733	3.65
CV%	173	131	106	106	57	18	8	39

LLOQ: below lower limit of quantification of 0.5 ng/mL;
[1]: value set to 0 for descriptive statistics and pharmacokinetic evaluation.

Table S10. Bioanalysis Data of Tetracaine in Skin after Single Topical Administration of 10 g/animal

Formulation	Animal numbers					
	1	2	3	Mean	SD	CV%
	Concentration (µg/g)					
Pliaglis (Period 2)	499	685	211	465	239	51
Z4T4L4 (Period 3)	34.0	67.0	37.2	46.1	18.2	40

Table S11. Bioanalysis Data of Lidocaine in Skin after Single Topical Administration of 10 g/animal

Formulation	Animal numbers					
	1	2	3	Mean	SD	CV%
	Concentration (µg/g)					
Pliaglis (Period 2)	306	600	169	358	220	61
Z4T4L4 (Period 3)	19.8	53.6	27.3	33.6	17.7	53

Table S12. Pharmacokinetic Parameters for Tetracaine after Single Intravenous Administration of 1 mg/kg Lidocaine HCl and Tetracaine HCl (Period 1)

Parameters		Animal numbers					
		1	2	3	Mean	SD	CV%
T_{last}	h	8	12	8	8-12$	n/a	n/a
C_0	ng/mL	112	148	3.79	87.8	75.0	85
AUC_{last}	h·ng/mL	77.5	77.9	19.5	58.3	33.6	58
AUC_{last}/Dose	h·kg·ng/mL/mg	77.5	77.9	19.5	58.3	33.6	58
AUC_{∞}	h·ng/mL	77.7	78.3	19.9	58.6	33.5	57
AUC_{∞}/Dose	h·kg·ng/mL/mg	77.7	78.3	19.9	58.6	33.5	57
% Extrapolated	%	0.3	0.5	2	n/a	n/a	n/a
λ_z	1/h	0.596	0.349	0.280	0.408	0.166	41
$T_{1/2}$	h	1.16	1.99	2.48	1.88	0.665	35
no. points		4	3	3	n/a	n/a	n/a
r^2		0.9093	0.9812	0.9809	n/a	n/a	n/a
Cl	mL/h/kg	12900	12800	50200	25300	21600	85
V_z	mL/kg	21600	36600	179000	79200	87100	110
V_{ss}	mL/kg	7710	13800	72100	31200	35500	114
MRT	h	0.599	1.08	1.44	1.04	0.420	40

n/a: not applicable; $: range; /Dose: dose-normalized to 1 mg/kg.

Table S13. Pharmacokinetic Parameters for Corrected Tetracaine after Single Intravenous Administration of 1 mg/kg Lidocaine HCl and Tetracaine HCl (Period 1)

Parameters		Animal numbers					
		1	2	3	Mean	SD	CV%
T_{last}	h	8	12	8	8-12[$]	n/a	n/a
C_0	ng/mL	158	243	4.64	135	121	89
AUC_{last}	h·ng/mL	106	108	23.8	79.3	48.1	61
AUC_{last}/Dose	h·kg·ng/mL/mg	106	108	23.8	79.3	48.1	61
AUC_{∞}	h·ng/mL	106	109	24.4	79.8	48.0	60
AUC_{∞}/Dose	h·kg·ng/mL/mg	106	109	24.4	79.8	48.0	60
% Extrapolated	%	0.4	0.3	2	n/a	n/a	n/a
λ_z	1/h	0.487	0.418	0.280	0.395	0.106	27
$T_{1/2}$	h	1.42	1.66	2.48	1.85	0.554	30
no. points		3	4	3	n/a	n/a	n/a
r^2		0.9553	0.9728	0.9809	n/a	n/a	n/a
Cl	mL/h/kg	9430	9190	41000	19900	18300	92
V_z	mL/kg	19300	22000	147000	62600	72700	116
V_{ss}	mL/kg	5550	9110	58900	24500	29800	122
MRT	h	0.589	0.991	1.44	1.01	0.424	42

n/a: not applicable; [$]: range; /Dose: dose-normalized to 1 mg/kg.

Table S14. Pharmacokinetic Parameters for Lidocaine after Single Intravenous Administration of 1 mg/kg Lidocaine HCl and Tetracaine HCl (Period 1)

Parameters		Animal numbers					
		1	2	3	Mean	SD	CV%
T_{last}	h	12	12	12	12	n/a	n/a
C_0	ng/mL	916	900	792	869	67.4	8
AUC_{last}	h·ng/mL	791	628	618	679	97.3	14
AUC_{last}/Dose	h·kg·ng/mL/mg	791	628	618	679	97.3	14
AUC_{∞}	h·ng/mL	794	630	619	681	98.0	14
AUC_{∞}/Dose	h·kg·ng/mL/mg	794	630	619	681	98.0	14
% Extrapolated	%	0.3	0.2	0.2	n/a	n/a	n/a
λ_z	1/h	0.386	0.460	0.441	0.429	0.0381	9
$T_{1/2}$	h	1.79	1.51	1.57	1.62	0.150	9
no. points		3	4	4	n/a	n/a	n/a
r^2		0.9940	0.9785	0.9819	n/a	n/a	n/a
Cl	mL/h/kg	1260	1590	1620	1490	198	13
V_z	mL/kg	3260	3450	3670	3460	204	6
V_{ss}	mL/kg	1450	1730	1640	1610	139	9
MRT	h	1.15	1.09	1.01	1.08	0.0712	7

n/a: not applicable; [$]: range; /Dose: dose-normalized to 1 mg/kg.

Table S15. Pharmacokinetic Parameters for Tetracaine after Single Topical Administration of 10 g/animal Pliaglis (Period 2)

Parameters		Animal numbers					
		1	2	3	Mean	SD	CV%
**Dose level (mg/kg)**		**33.7**	**40.0**	**42.9**			
T_{last}	h	24	24	24	24	n/a	n/a
T_{max}	h	4	1	8	1-8$^{\$}$	n/a	n/a
C_{max}	ng/mL	3.04	6.45	0.835	3.44	2.83	82
AUC_{last}	h·ng/mL	32.7	38.7	13.4	28.3	13.2	47
AUC_{last}/Dose	h·kg·ng/mL/mg	0.971	0.967	0.313	0.750	0.378	50
AUC_{∞}	h·ng/mL	38.0	41.1	n/c	39.6	ID	ID
AUC_{∞}/Dose	h·kg·ng/mL/mg	1.13	1.03	n/c	1.08	ID	ID
% Extrapolated	%	14	6	n/c	n/a	n/a	n/a
λ_z	1/h	0.0901	0.137	n/c	0.114	ID	ID
$T_{1/2}$	h	7.69	5.06	n/c	6.37	ID	ID
No.points		3	3	n/c	n/a	n/a	n/a
r^2		0.8138	0.9607	n/c	n/a	n/a	n/a
F	%	1.25	1.24	1.61	1.37	0.207	15

n/a: not applicable; $^{\$}$: range; n/c: could not be calculated; ID: insufficient Data;
/Dose: dose-normalized to 1 mg/kg.

Table S16. Pharmacokinetic Parameters for Corrected Tetracaine after Single Topical Administration of 10 g/animal Pliaglis (Period 2)

Parameters		Animal numbers					
		1	2	3	Mean	SD	CV%
**Dose level (mg/kg)**		**33.7**	**40.0**	**42.9**			
T_{last}	h	24	24	24	24	n/a	n/a
T_{max}	h	4	1	8	1-8$^{\$}$	n/a	n/a
C_{max}	ng/mL	3.73	7.90	1.02	4.22	3.46	82
AUC_{last}	h·ng/mL	40.1	47.4	16.5	34.6	16.2	47
AUC_{last}/Dose	h·kg·ng/mL/mg	1.19	1.18	0.384	0.919	0.464	50
AUC_{∞}	h·ng/mL	46.6	50.3	n/c	48.5	ID	ID
AUC_{∞}/Dose	h·kg·ng/mL/mg	1.38	1.26	n/c	1.32	ID	ID
% Extrapolated	%	14	6	n/c	n/a	n/a	n/a
λ_z	1/h	0.0901	0.137	n/c	0.114	ID	ID
$T_{1/2}$	h	7.69	5.06	n/c	6.37	ID	ID
No.points		3	3	n/c	n/a	n/a	n/a
r^2		0.8138	0.9607	n/c	n/a	n/a	n/a
F	%	1.12	1.08	1.57	1.26	0.273	22

n/a: not applicable; $^{\$}$: range; n/c: could not be calculated; ID: insufficient Data; NR: not reported;
/Dose: dose-normalized to 1 mg/kg.

Table S17. Pharmacokinetic Parameters for Lidocaine after Single Topical Administration of 10 g/animal Pliaglis (Period 2)

Parameters		Animal numbers					
		1	2	3	Mean	SD	CV%
**Dose level (mg/kg)**		**33.7**	**40.0**	**42.9**			
T_{last}	h	24	24	24	24	n/a	n/a
T_{max}	h	2	4	4	2-4$^{\$}$	n/a	n/a
C_{max}	ng/mL	56.5	21.4	43.7	40.6	17.8	44
AUC_{last}	h·ng/mL	468	270	374	371	99.2	27
AUC_{last}/Dose	h·kg·ng/mL/mg	13.9	6.74	8.73	9.78	3.69	38
AUC_{∞}	h·ng/mL	525	311	NR	418	ID	ID
AUC_{∞}/Dose	h·kg·ng/mL/mg	15.6	7.77	NR	11.7	ID	ID
% Extrapolated	%	11	13	NR	n/a	n/a	n/a
λ_z	1/h	0.103	0.0977	NR	0.0825	ID	ID
$T_{1/2}$	h	6.71	7.10	NR	9.58	ID	ID
No.points		4	3	NR	n/a	n/a	n/a
r^2		0.8146	0.9550	NR	n/a	n/a	n/a
F	%	1.76	1.07	1.41	1.41	0.342	24

n/a: not applicable; $^{\$}$: range; n/c: could not be calculated; ID: insufficient Data; NR: not reported; /Dose: dose-normalized to 1 mg/kg.

Table S18. Pharmacokinetic Parameters for Tetracaine after Single Topical Administration of 10 g/animal Z4T4L4 (Period 3)

Parameters		Animal numbers					
		1	2	3	Mean	SD	CV%
**Dose level (mg/kg)**		**18.2**	**21.1**	**23.4**			
T_{last}	h	24	24	24	24	n/a	n/a
T_{max}	h	2	4	8	2-8$^{\$}$	n/a	n/a
C_{max}	ng/mL	3.55	3.07	1.75	2.79	0.929	33
AUC_{last}	h·ng/mL	34.4	47.8	22.3	34.8	12.7	37
AUC_{last}/Dose	h·kg·ng/mL/mg	1.89	2.26	0.954	1.70	0.675	40
AUC_{∞}	h·ng/mL	NR	NR	n/c	n/a	n/a	n/a
AUC_{∞}/Dose	h·kg·ng/mL/mg	NR	NR	n/c	n/a	n/a	n/a
% Extrapolated	%	NR	NR	n/c	n/a	n/a	n/a
λ_z	1/h	NR	NR	n/c	n/a	n/a	n/a
$T_{1/2}$	h	NR	NR	n/c	n/a	n/a	n/a
No.points		NR	NR	n/c	n/a	n/a	n/a
r^2		NR	NR	n/c	n/a	n/a	n/a
F	%	2.44	2.90	4.89	3.41	1.30	38

n/a: not applicable; $^{\$}$: range; n/c: could not be calculated; ID: insufficient Data; NR: not reported; /Dose: dose-normalized to 1 mg/kg.

Table S19. Pharmacokinetic Parameters for Corrected Tetracaine after Single Topical Administration of 10 g/animal Z4T4L4 (Period 3)

Parameters		Animal numbers					
		1	2	3	Mean	SD	CV%
**Dose level (mg/kg)**		**18.2**	**21.1**	**23.4**			
T_{last}	h	24	24	24	24	n/a	n/a
T_{max}	h	2	4	8	2-8$	n/a	n/a
C_{max}	ng/mL	4.34	3.76	2.15	3.41	1.14	33
AUC_{last}	h·ng/mL	42.9	60.8	28.4	44.1	16.2	37
AUC_{last}/Dose	h·kg·ng/mL/mg	2.36	2.88	1.22	2.15	0.853	40
AUC_{∞}	h·ng/mL	NR	n/c	n/c	n/a	n/a	n/a
AUC_{∞}/Dose	h·kg·ng/mL/mg	NR	n/c	n/c	n/a	n/a	n/a
% Extrapolated	%	NR	n/c	n/c	n/a	n/a	n/a
λ_z	1/h	NR	n/c	n/c	n/a	n/a	n/a
$T_{1/2}$	h	NR	n/c	n/c	n/a	n/a	n/a
No.points		NR	n/c	n/c	n/a	n/a	n/a
r^2		NR	n/c	n/c	n/a	n/a	n/a
F	%	2.23	2.67	5.13	3.34	1.56	47

n/a: not applicable; $: range; n/c: could not be calculated; ID: insufficient Data; NR: not reported; /Dose: dose-normalized to 1 mg/kg.

Table S20. Pharmacokinetic Parameters for Lidocaine after Single Topical Administration of 10 g/animal Z4T4L4 (Period 3)

Parameters		Animal numbers					
		1	2	3	Mean	SD	CV%
**Dose level (mg/kg)**		**18.2**	**21.1**	**23.4**			
T_{last}	h	24	24	24	24	n/a	n/a
T_{max}	h	2	4	8	2-8$	n/a	n/a
C_{max}	ng/mL	36.4	16.3	16.5	23.1	11.6	50
AUC_{last}	h·ng/mL	347	230	258	278	61.3	22
AUC_{last}/Dose	h·kg·ng/mL/mg	19.1	10.9	11.0	13.7	4.69	34
AUC_{∞}	h·ng/mL	NR	NR	n/c	n/a	n/a	n/a
AUC_{∞}/Dose	h·kg·ng/mL/mg	NR	NR	n/c	n/a	n/a	n/a
% Extrapolated	%	NR	NR	n/c	n/a	n/a	n/a
λ_z	1/h	NR	NR	n/c	n/a	n/a	n/a
$T_{1/2}$	h	NR	NR	n/c	n/a	n/a	n/a
No.points		NR	NR	n/c	n/a	n/a	n/a
r^2		NR	NR	n/c	n/a	n/a	n/a
F	%	2.41	1.74	1.78	1.98	0.380	19

n/a: not applicable; $: range; n/c: could not be calculated; ID: insufficient Data; NR: not reported; /Dose: dose-normalized to 1 mg/kg.

Table S21. Stability of Tetracaine in Göttingen Minipig Plasma with and without Acidification: Peak Areas, Calculated Concentrations, and Accuracy over 0–1.5 Hours at 0–4 °C

Sample Name	Sample Type	Analyte Peak Name	Analyte Peak Area (counts)	Analyte Concentration (ng/mL)	Area Ratio	IS Peak Area (counts)	Use Record	Calculated Concentration (ng/mL)	Accuracy (%)	Average Area Ratio of triplicate	Area ratio of T = 0 (%)	Remarks
DB	Double Blank	Tetracaine	0	0	-	7	0	N/A	N/A			
DB	Double Blank	Tetracaine	0	0	-	7	0	N/A	N/A			
SB	Blank	Tetracaine	0	0	0	806900	N/A	N/A				
SB	Blank	Tetracaine	0	0	0	785200	N/A	N/A				
Qc mid_No FA_0-hours-1	Quality Control	Tetracaine	51550	8	0.07312	705100	1	0	N/A			
Qc mid_No FA_0-hours-2	Quality Control	Tetracaine	50920	8	0.07276	699800	1	0	N/A	0.0727	100	non-acidified T = 0 hours
Qc mid_No FA_0-hours-3	Quality Control	Tetracaine	49560	8	0.07235	685000	1	0	N/A			
Qc mid_+ FA_0-hours-1	Quality Control	Tetracaine	74050	8	0.08946	827800	1	0	N/A			
Qc mid_+ FA_0-hours-2	Quality Control	Tetracaine	67210	8	0.0886	758700	1	0	N/A	0.0910	100	acidified T = 0 hours
Qc mid_+ FA_0-hours-3	Quality Control	Tetracaine	76250	8	0.0948	804300	1	0	N/A			
SB_No FA	Blank	Tetracaine	0	0	0	739200	N/A	N/A				

SB_+ FA	Blank	Tetracaine	0	0	0	747500	N/A	N/A				
Qc mid_No FA_0.5-hours-1	Quality Control	Tetracaine	46510	8	0.05699	816100	1	0	N/A			
Qc mid_No FA_0.5-hours-2	Quality Control	Tetracaine	46220	8	0.05978	773200	1	0	N/A	0.0595	81.7	non-acidified T = 0.5 hours
Qc mid_No FA_0.5-hours-3	Quality Control	Tetracaine	45030	8	0.06162	730800	1	0	N/A			
Qc mid_+ FA_0.5-hours-1	Quality Control	Tetracaine	62600	8	0.09203	680200	1	0	N/A			
Qc mid_+ FA_0.5-hours-2	Quality Control	Tetracaine	68010	8	0.09076	749400	1	0	N/A	0.0917	101	acidified T = 0.5 hours
Qc mid_+ FA_0.5-hours-3	Quality Control	Tetracaine	65870	8	0.09234	713300	1	0	N/A			
SB_No FA	Blank	Tetracaine	0	0	0	727000	N/A	N/A				
SB_+ FA	Blank	Tetracaine	0	0	0	700200	N/A	N/A				
Qc mid_No FA_1.0-hours-1	Quality Control	Tetracaine	34010	8	0.05117	664600	1	0	N/A			
Qc mid_No FA_1.0-hours-2	Quality Control	Tetracaine	38650	8	0.05314	727400	1	0	N/A	0.0514	70.6	non-acidified T = 1.0 hours
Qc mid_No FA_1.0-hours-3	Quality Control	Tetracaine	34650	8	0.0498	695900	1	0	N/A			
Qc mid_+ FA_1.0-hours-1	Quality Control	Tetracaine	69550	8	0.08837	787000	1	0	N/A			
Qc mid_+ FA_1.0-hours-2	Quality Control	Tetracaine	70020	8	0.08987	779100	1	0	N/A	0.0917	101	acidified T = 1.0 hours

Qc mid_+ FA_1.0-hours-3	Quality Control	Tetracaine	73930	8	0.09693	762700	1	0	N/A			
SB_No FA	Blank	Tetracaine	0	0	0	766000	N/A	N/A				
SB_+ FA	Blank	Tetracaine	0	0	0	826700	N/A	N/A				
Qc mid_No FA_1.5-hours-1	Quality Control	Tetracaine	36620	8	0.04793	764000	1	0	N/A			
Qc mid_No FA_1.5-hours-1	Quality Control	Tetracaine	32490	8	0.0452	718800	1	0	N/A	0.0469	64.5	non-acidified T = 1.5 hours
Qc mid_No FA_1.5-hours-1	Quality Control	Tetracaine	35440	8	0.04766	743600	1	0	N/A			
Qc mid_+ FA_1.5-hours-1	Quality Control	Tetracaine	67480	8	0.08909	757400	1	0	N/A			
Qc mid_+ FA_1.5-hours-2	Quality Control	Tetracaine	75320	8	0.09271	812400	1	0	N/A	0.0930	102	acidified T = 1.5 hours
Qc mid_+ FA_1.5-hours-3	Quality Control	Tetracaine	76830	8	0.09713	791000	1	0	N/A			
SB_No FA	Blank	Tetracaine	0	0	0	813700	N/A	N/A				
SB_+ FA	Blank	Tetracaine	0	0	0	743500	N/A	N/A				

Legend: Plasma quality control (QC) samples containing tetracaine (8 ng/mL) were analyzed with and without acidification using 1% formic acid (FA). Samples were stored on ice (0–4 °C) for up to 1.5 h. Non-acidified samples showed progressive degradation: 81.7% recovery at 0.5 h and 70.6% recovery at 1.0 h. Based on these stability data, correction factors were calculated as the inverse of recovery (1/Recovery) to reconstruct plasma concentrations: a factor of 1.22 was applied for samples stored up to 0.5 h (17–44 min), and a factor of 1.42 for samples stored up to 1.0 h (45–65 min). Acidified samples remained stable (101–102%), confirming the efficacy of the stabilization protocol.
